# Network pharmacology-based and molecular docking-based analysis of You-Gui-Yin for the treatment of osteonecrosis of the femoral head

**DOI:** 10.1097/MD.0000000000035581

**Published:** 2023-10-27

**Authors:** Zhi-Yuan Yao, Shu-Yao Fan, Zhou-Feng Song, Zhan-Chun Li

**Affiliations:** a Department of Orthopedics, The First Affiliated Hospital of Zhejiang Chinese Medical University (Zhejiang Provincial Hospital of Traditional Chinese Medicine), Hangzhou Economic and Technological Development Zone, Hangzhou, Zhejiang, China; b Department of Breast Surgery, The First Affiliated Hospital of Zhejiang Chinese Medical University (Zhejiang Provincial Hospital of Traditional Chinese Medicine), Hangzhou Economic and Technological Development Zone, Hangzhou, Zhejiang, China; c Department of Orthopedics, The First Affiliated Hospital of Zhejiang Chinese Medical University (Zhejiang Provincial Hospital of Traditional Chinese Medicine), Hangzhou Economic and Technological Development Zone, Hangzhou, Zhejiang, China; d Department of Orthopedics, The First Affiliated Hospital of Zhejiang Chinese Medical University (Zhejiang Provincial Hospital of Traditional Chinese Medicine), Hangzhou Economic and Technological Development Zone, Hangzhou, Zhejiang, China.

**Keywords:** molecular docking, network pharmacology, osteonecrosis of the femoral head, traditional Chinese medicine, You-Gui-Yin

## Abstract

You-Gui-Yin (YGY) is a classic prescription for warming up kidney-Yang and filling in kidney essence in traditional Chinese medicine, and has been used to treat osteonecrosis of the femoral head (ONFH) effectively. However, the underlying mechanisms are still unknown. This study is aimed at exploring the possible mechanisms of action of the YGY in the treatment of ONFH based on network pharmacology and molecular docking. TCMSP was used to screen the active components and targets of YGY. The disease targets of ONFH were collected in several public databases. The protein-protein interaction (PPI) Network was constructed using the STRING platform. The Metascape database platform was used for Gene Ontology (GO) and Kyoto Encyclopedia of Genes and Genomes (KEGG) analyses. The key active components and core target proteins of YGY in the treatment of ONFH were verified by the molecular docking. 120 active components were obtained from YGY, among which 73 components were hit by the 117 drug-disease intersection targets. Key effective components included quercetin, kaempferol, beta-sitosterol, glycitein, beta-carotene, and so on. Core target proteins included ALB, AKT1, TNF, IL6, TP53, and so on. According to GO and KEGG analyses, there were 1762 biological processes, 94 cellular component, 138 molecular function and 187 signaling pathways involved. we selected the top 20 biological processes (BP), cellular components (CC), molecular functions (MF) and signaling pathways to draw the heat maps, showing that Lipid and atherosclerosis signaling pathway, IL-17 signaling pathway, HIF-1 signaling pathway, relaxin signaling pathway and MAPK signaling pathway and other pathways may play a key role in the treatment of ONFH by YGY. The results of molecular docking showed that key effective components and corresponding core target proteins exhibited the good binding activity. YGY can treat ONFH through multicomponents, multitargets, and multipathways, which provides a reference for the subsequent research, development of targeted drugs and clinical application.

## 1. Introduction

Osteonecrosis of the femoral head (ONFH) which leads to chronic pain, femoral head collapse and dysfunction of the hip joint is one of the outstanding problems in the field of orthopedics and difficult to treat. Its etiology and pathogenesis are still unclear and complex, and current theories related to etiology and pathogenesis involve in trauma, corticosteroids, alcohol use, vascular thrombosis, blood dyscrasias and miscellaneous factors.^[1]^ In the terminal stage of the disease, most patients must undergo artificial hip replacement.^[2]^ However, artificial hip replacement will make great damage to normal tissues, high economic cost, certain surgical risks and postoperative complications, and patients may still need to undergo another or even multiple revision after the operation. Therefore, there is an urgent need to find the effective conservative treatment in the early and middle stages of the disease.

You-Gui-Yin (YGY), derived from *Jingyue Complete Works*, is composed of 8 traditional Chinese medicines (TCM): Shudihuang (Radix Rehmanniae Praeparata), Shanyao (Dioscorea opposite), Shanzhuyu (Cornus officinalis Siebold & Zucc.), Gouqi (Lycium chinense Mill.), Duzhong (Eucommia ulmoides Oliv.), Fuzi (Radix Aconiti Late-ralis Preparata), Gancao (Glycyrrhiza uralensis Fisch.) and Rougui (Cinnamomum cassia Presl.). Among them, Radix Aconiti Late-ralis Preparata and Cinnamomum cassia Presl warm and tonify the kidney-Yang, known as monarch drugs in the prescription; Radix Rehmanniae Praeparata, Dioscorea opposita, Cornus officinalis Siebold & Zucc. and Lycium chinense Mill. nourish the kidney-Yin, enrich essence and marrow, nourish liver and strengthen spleen, take the meaning of “seeking Yang in the Yin,” known as ministerial drugs; Eucommia ulmoides Oliv. nourishes liver and kidney, strengthens bones and muscles, known as the adjuvant drug; Glycyrrhiza uralensis Fisch. reconciles the medicines, known as conductant drug. It is a classic prescription for warming up kidney-Yang and filling in kidney essence in traditional Chinese medicine, and there is a synergistic effect between Yang tonic and Yin tonic to obviously improve or even correct the “kidney-Yang deficiency” state.^[3,4]^ On the other hand, ONFH is often be differentiated by traditional Chinese medicine (TCM) as the “kidney-Yang deficiency” state.^[5]^ So YGY is a kind of effective prescription for treating ONFH, and many clinical and animal experiments have proved that YGY is effective in treating ONFH.^[6–8]^ However, as YGY is a compound preparation, the mechanism of YGY in ONFH treatment has not been fully understood. Therefore, the article selected the monarch drugs, ministerial drugs and adjuvant drug (Radix Rehmanniae Praeparata, Dioscorea opposita, Cornus officinalis Siebold & Zucc., Lycium chinense Mill., Eucommia ulmoides Oliv., Radix Aconiti Late-ralis Preparata and Cinnamomum cassia Presl.) which played the major role in the prescription for further study.

The occurrence and development of diseases in the human body are caused by the interaction of many comprehensive factors. Therefore, the effective treatment of many diseases requires multi-target and multi-pathway therapeutic regimens. The concept of network pharmacology which analyzes the interaction between the drug, disease and targets, proposed by Hopkins in 2007, offers a multi-pathway, multi-target approach to evaluate the intervention or effect of drugs on diseases.^[9]^ The application of this research method in the analysis of TCM prescription is in line with the characteristics of overall compatibility of TCM prescription and the comprehensive multi-way therapy of diseases. Molecular docking is a computer-aided theoretical simulation method based on the “lock and key principle” of ligand-receptor interaction, which can be used to simulate the interaction between small molecular ligands of drug and biological target proteins in body and predict their binding mode and affinity.^[10,11]^ This effect is helpful for screening drugs, explaining the cause of the activity of drug molecules and optimizing the molecular structure of drugs.

**Figure 1.**
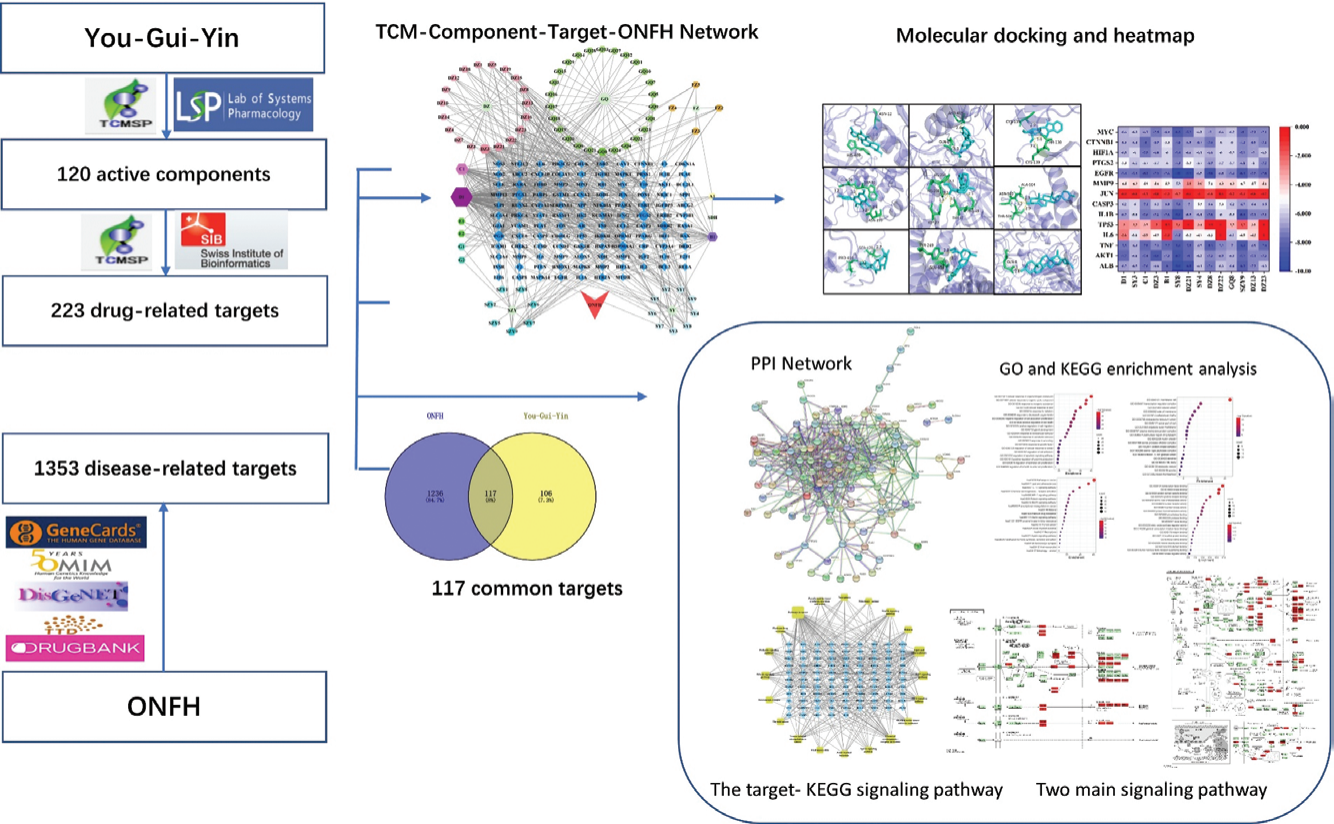
Workflow of network pharmacology and molecular docking.

In this study, network pharmacology and molecular docking techniques were used to find and verify the active components, action targets, biological processes, cellular components, molecular functions and signaling pathways of YGY in ONFH treatment. This study aims to explore the potential mechanism of YGY in the treatment of ONFH, and further deepen the understanding of the pharmacological mechanism and clinical application of this prescription. The flowchart of the study design is shown in Figure 1.

## 2. Methods

### 2.1. Screening the active components and targets for You-Gui-Yin

The active components of Radix Rehmanniae Praeparata(SDH), Dioscorea opposita(SY), Cornus officinalis Siebold & Zucc. (SZY), Lycium chinense Mill.(GQ), Eucommia ulmoides Oliv.(DZ), Radix Aconiti Late-ralis Preparata(FZ), and Cinnamomum cassia Presl(RG). were acquired from the new edition of Traditional Chinese Medicine Database and Analysis Platform (TCMSP) (https://tcmsp-e.com/). According to the standards of Oral bioavailability (OB) ≥ 30% and drug-likeness (DL) ≥ 0.18, we screened the active components of You-Gui-Yin in the TCMSP database and then obtained the corresponding targets for each active component. Meanwhile, other active components not retrieved were supplemented by related literature reviews. If the targets of active components cannot be obtained from the TCMSP database, the prediction of the relevant targets was conducted in Swiss Target Prediction (http://www.swisstargetprediction.ch). In order to standardize the all identified targets of active components, we put the targets into the UniProt database (https://www.uniprot.org/) and set the protein type as “Homo sapiens.”

### 2.2. Screening of predictive targets related to ONFH

The search for target genes related to the disease was obtained using the keywords “osteonecrosis of the femoral head,” “femur head necrosis,” “avascular necrosis of femur head” and “femoral head necrosis” in the the OMIM (https://omim.org/), GeneCards (https://www.genecards.org/), DisGeNET (https://www.disgenet.org/), DigSee (http://gcancer.org/digsee/), TTD (http://db.idrblab.net/ttd/) and Drugbank (https://go.Drugbank.com/) database search. These target genes were standardized through Uniprot database, and then the disease targets obtained from above databases were integrated to get the total target genes of ONFH after deleting the repeated ones.

### 2.3. Acquisition of common targets between drug components and diseases

The targets of active components of YGY and ONFH were introduced into online Venny2.1 platform (https://bioinfogp.cnb.csic.es/tools/venny/) to get the intersection targets and construct Venn diagram.

### 2.4. Construction of TCM-component-target-ONFH network

Compositions of YGY, active components, intersecting targets, and ONFH were introduced into Cytoscape 3.8.2 software, and the Network of “TCM-component-target-ONFH” was constructed to visualize the result analysis. Then the key active components were screened according to “Degree” value.

### 2.5. Construction of the protein-protein interaction (PPI) network

The intersection targets of the active components of YGY and ONFH were imported into the STRING database (version11.5, https://string-db.org/), the minimum required interaction score was set to “highest confidence>0.9.” The species was selected as “Homo sapiens,” the disconnected nodes were hid in the network and the other settings were default to construct the protein-protein interaction (PPI) Network. Then the core target proteins were screened according to “Degree” value.

### 2.6. GO and KEGG pathway enrichment analyses of the intersecting targets

The Metascape database platform (https://metascape.org/gp/index.html#/main/step1) was used for Gene Ontology (GO) functional enrichment analysis and Kyoto Encyclopedia of Genes and Genomes (KEGG) signaling pathway enrichment by importing the intersection targets into this database. Results with values of *P* < .01 were selected, and “Homo species” was defined as the species. Finally, we screened out the first 20 categories of biological processes (BP), cellular components (CC), molecular functions (MF) and KEGG signal pathways, respectively. Then the bioinformatic online platform (https://www.bioinformatics.com.cn/) was used to visualize the result analyses as bubble charts. Based on the data obtained from KEGG signaling pathway enrichment analysis, the target- KEGG signaling pathway network was constructed by introducing intersecting targets related to the pathways and core signaling pathways into Cytoscape3.8.2 software. The KEGG Mapper tool (https://www.genome.jp/kegg/mapper/) was used to visualize the signaling pathway.

### 2.7. Molecular docking and verification of key active components with core target proteins

In order to further clarify and verify the internal molecular mechanism of YGY in treating ONFH, the 14 key active components and 14 core target proteins screened from above methods (mentioned in 2.4 and 2.5) were used for molecular docking. The 2D structures of the potential active components (*Mol2 format) in YGY were downloaded from the TCMSP database while the 3D structures of the target proteins (*PDB format) treated by YGY in ONFH were downloaded from the RCSB PDB database (https://www.rcsb.org/).

The PyMol 2.5.2 software (https://www.pymol.org/) was used to remove waters and ligands from the target protein receptors. And AutoDock Tools was used to hydrogenate the receptors, calculate the charge and other pretreatments. The center coordinate and size of the box were set based on the position of the active site of target protein receptors and the area where the ligands might bind. Molecular docking of the receptors and ligands was conducted by AutoDock Vina. Affinity was the binding energy for the molecular docking, and when the affinity score value was lower, the binding affinity between the receptor and the ligand was stronger. The affinity score value ≤ 0 kcal/mol indicated that the active component could bind and interact with the target protein, whereas affinity score value <−5 kcal/mol demonstrated a high binding activity and strong force. The binding energy results obtained from the analysis were imported into Origin 2021 software (https://www.originlab.com/) to create 2D heat map. Finally, the docking models were visualized using the PyMol software (https://www.pymol.org/).

## 3. Results

### 3.1. The active components and predictive targets for You-Gui-Yin

With the criteria of OB ≥ 30% and DL ≥ 0.18, a total of 132 active components were screened by searching the TCMSP database, including 2 from Radix Rehmanniae Praeparata, 16 from Dioscorea opposita, 20 from Cornus officinalis Siebold & Zucc., 45 from Lycium chinense Mill., 28 from Eucommia ulmoides Oliv., 21 from Radix Aconiti Late-ralis Preparata and 0 from Cinnamomum cassia Presl. As the Cinnamomum cassia Presl had no active component selected, the article no longer considered its effect. After eliminating overlapping active components, 120 active compounds were remaining.

A total of 1194 drug targets were screened using the TCMSP database and were standardized by the UniProt database, which included the 33 from Radix Rehmanniae Praeparata, 138 from Dioscorea opposita, 129 from Cornus officinalis Siebold & Zucc., 356 from Lycium chinense Mill., 508 from Eucommia ulmoides Oliv., 30 from Radix Aconiti Late-ralis Preparata. After removing repeated targets, 223 targets and their abbreviations were obtained.

### 3.2. The predictive targets related to ONFH

OMIM, GeneCards, DisGeNET, DigSee, TTD, and Drugbank databases were used to search for “osteonecrosis of the femoral head,” “femur head necrosis,” “avascular necrosis of femur head” and “femoral head necrosis” related targets. After removing the duplicate targets, a total of 1353 target genes associated with ONFH were obtained.

### 3.3. Common targets between drug components and diseases

**Figure 2.**
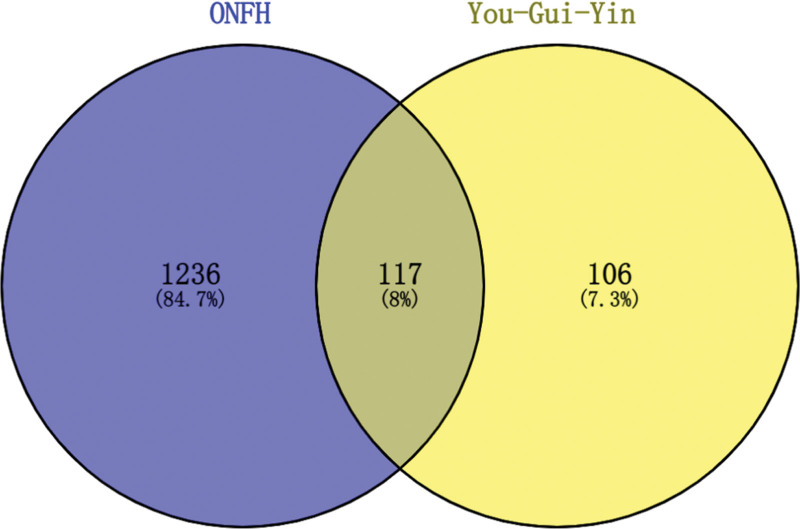
Venn diagram of drug targets and disease targets. The blue circle indicates the targets of ONFH, the yellow circle indicates the targets of YGY, and the intersecting part of the 2 circles indicates the common targets of both. YGY = You-Gui-Yin, ONFH = Osteonecrosis of the femoral head.

The 117 drug-disease intersection targets (the potential targets of YGY in ONFH treatment, Table 1) were obtained by intersecting the 223 drug targets with 1353 disease targets using the Venny online mapping platform (Fig. 2). Notably, 73 active components corresponded to the 117 potential targets. They were the effective components of YGY in ONFH treatment (Table 1).

**Table 1:** The total effective components of YGY in the treatment of ONFH.

Number	Mol ID	Molecule name	OB (%)	DL
A1	MOL000359	sitosterol	36.91	0.75
B1	MOL000449	Stigmasterol	43.83	0.76
C1	MOL000358	beta-sitosterol	36.91	0.75
D1	MOL000098	quercetin	46.43	0.28
E1	MOL005438	campesterol	37.58	0.71
E2	MOL000953	CLR	37.87	0.68
G1	MOL001494	Mandenol	42	0.19
G2	MOL001495	Ethyl linolenate	46.1	0.2
DZ1	MOL002058	40957-99-1	57.2	0.62
DZ2	MOL000211	Mairin	55.38	0.78
DZ3	MOL000422	kaempferol	41.88	0.24
DZ4	MOL004367	olivil	62.23	0.41
DZ5	MOL000443	Erythraline	49.18	0.55
DZ7	MOL006709	AIDS214634	92.43	0.55
DZ8	MOL007059	3-beta-Hydroxymethyllenetanshiquinone	32.16	0.41
DZ9	MOL000073	ent-Epicatechin	48.96	0.24
DZ10	MOL007563	Yangambin	57.53	0.81
DZ12	MOL009009	(+)-medioresinol	87.19	0.62
DZ13	MOL009015	(-)-Tabernemontanine	58.67	0.61
DZ14	MOL009027	Cyclopamine	55.42	0.82
DZ15	MOL009029	Dehydrodiconiferyl alcohol 4,gamma’-di-O-beta-D-glucopyanoside_qt	51.44	0.4
DZ16	MOL009031	Cinchonan-9-al, 6’-methoxy-, (9R)-	68.22	0.4
DZ18	MOL009047	(+)-Eudesmin	33.29	0.62
DZ19	MOL009053	4-[(2S,3R)-5-[(E)-3-hydroxyprop-1-enyl]-7-methoxy-3-methylol-2,3-dihydrobenzofuran-2-yl]-2-methoxy-phenol	50.76	0.39
DZ21	MOL002773	beta-carotene	37.18	0.58
DZ22	MOL008240	(E)-3-[4-[(1R,2R)-2-hydroxy-2-(4-hydroxy-3-methoxy-phenyl)-1-methylol-ethoxy]-3-methoxy-phenyl]acrolein	56.32	0.36
DZ23	MOL011604	Syringetin	36.82	0.37
FZ2	MOL002388	Delphin_qt	57.76	0.28
FZ3	MOL002392	Deltoin	46.69	0.37
FZ4	MOL002395	Deoxyandrographolide	56.3	0.31
FZ5	MOL002398	Karanjin	69.56	0.34
GQ1	MOL001323	Sitosterol alpha1	43.28	0.78
GQ3	MOL001979	LAN	42.12	0.75
GQ4	MOL005406	Atropine	45.97	0.19
GQ5	MOL006209	Cyanin	47.42	0.76
GQ6	MOL007449	24-methylidenelophenol	44.19	0.75
GQ7	MOL008173	daucosterol_qt	36.91	0.75
GQ8	MOL008400	Glycitein	50.48	0.24
GQ9	MOL009604	14b-pregnane	34.78	0.34
GQ10	MOL009617	24-ethylcholest-22-enol	37.09	0.75
GQ11	MOL009618	24-ethylcholesta-5,22-dienol	43.83	0.76
GQ12	MOL009620	24-methyl-31-norlanost-9(11)-enol	38	0.75
GQ13	MOL009621	24-methylenelanost-8-enol	42.37	0.77
GQ14	MOL009622	Fucosterol	43.78	0.76
GQ15	MOL009633	31-norlanost-9(11)-enol	38.35	0.72
GQ16	MOL009634	31-norlanosterol	42.2	0.73
GQ17	MOL009635	4,24-methyllophenol	37.83	0.75
GQ18	MOL009639	Lophenol	38.13	0.71
GQ19	MOL009640	4alpha,14alpha,24-trimethylcholesta-8,24-dienol	38.91	0.76
GQ20	MOL009641	4alpha,24-dimethylcholesta-7,24-dienol	42.65	0.75
GQ21	MOL009642	4alpha-methyl-24-ethylcholesta-7,24-dienol	42.3	0.78
GQ22	MOL009644	6-Fluoroindole-7-Dehydrocholesterol	43.73	0.72
GQ23	MOL009646	7-O-Methylluteolin-6-C-beta-glucoside_qt	40.77	0.3
GQ24	MOL009650	Atropine	42.16	0.19
GQ27	MOL009677	lanost-8-en-3beta-ol	34.23	0.74
GQ28	MOL009678	lanost-8-enol	34.23	0.74
GQ29	MOL009681	Obtusifoliol	42.55	0.76
SY1	MOL001559	piperlonguminine	30.71	0.18
SY2	MOL001736	(-)-taxifolin	60.51	0.27
SY3	MOL000322	Kadsurenone	54.72	0.38
SY4	MOL005430	hancinone C	59.05	0.39
SY5	MOL005435	24-Methylcholest-5-enyl-3belta-O-glucopyranoside_qt	37.58	0.72
SY6	MOL005440	Isofucosterol	43.78	0.76
SY7	MOL005458	Dioscoreside C_qt	36.38	0.87
SY8	MOL000546	Diosgenin	80.88	0.81
SY9	MOL005465	AIDS180907	45.33	0.77
SZY1	MOL001771	poriferast-5-en-3beta-ol	36.91	0.75
SZY2	MOL002879	Diop	43.59	0.39
SZY6	MOL005503	Cornudentanone	39.66	0.33
SZY5	MOL005481	2,6,10,14,18-pentamethylicosa-2,6,10,14,18-pentaene	33.4	0.24
SZY7	MOL005530	Hydroxygenkwanin	36.47	0.27
SZY8	MOL005531	Telocinobufagin	69.99	0.79
SZY9	MOL008457	Tetrahydroalstonine	32.42	0.81

DL = drug-likeness, OB = oral bioavailability, ONFH = osteonecrosis of the femoral head, YGY = You-Gui-Yin.

### 3.4. TCM-component-target-ONFH network construction

**Figure 3.**
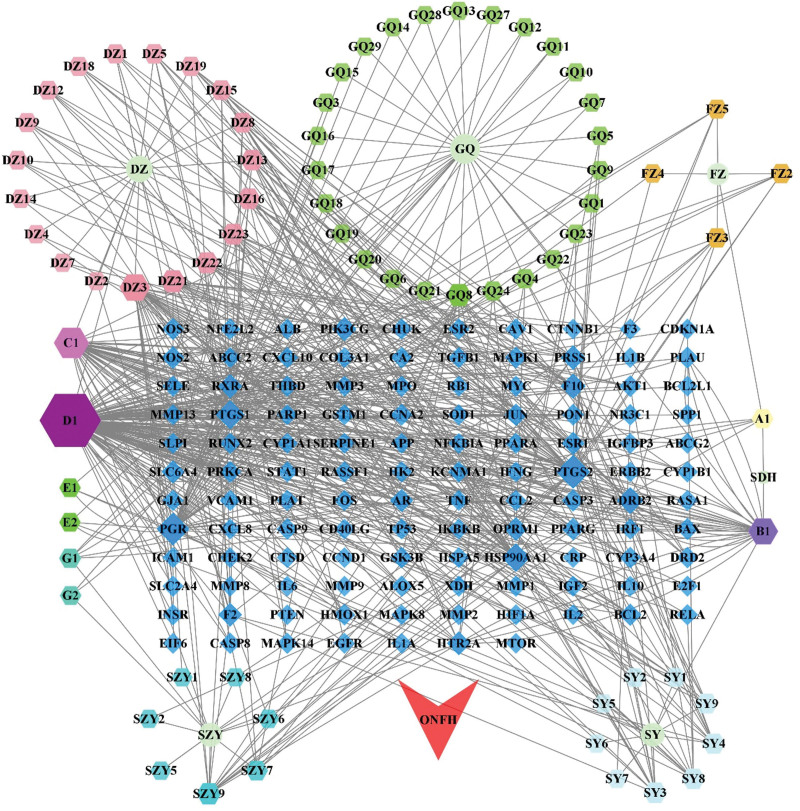
TCM-component-target-ONFH network. The red sagittate node represent the disease “ONFH,” the 117 blue rhombic nodes represent the drug-disease intersection target genes, the 6 circular nodes denote the 6 different kinds of Traditional Chinese Medicine in YGY, the 73 hexagonal nodes denote the drug effective components. TCM = traditional Chinese medicines, ONFH = Osteonecrosis of the femoral head, YGY = You-Gui-Yin.

Cytoscape 3.8.2 was used to construct the TCM-component-target-ONFH network of YGY for ONFH treatment involving 197 nodes (including 117 target genes, 73 drug effective components, 6 TCM, and 1 disease name) and 613 edges (Fig. 3). The red sagittate node represented the disease “ONFH,” the 117 blue rhombic nodes represented the drug-disease intersection target genes, the 6 circular nodes denoted the 6 different kinds of Traditional Chinese Medicine in YGY, the 73 hexagonal nodes denoted the drug effective components. We calculated the degree values of the network between 117 target genes and 73 drug effective components. And the node sizes depended on degree value. The larger the node was, the larger the degree value was. A higher degree implied that the effective component played a more critical role in the network. The effective components with the top 14-degree values were selected as the key effective components of YGY in ONFH treatment (Table 2).

**Table 2:** The key effective components of YGY in the treatment of ONFH.

Number	Source	Mol ID	Molecule name	OB(%)	DL	Degree
D1	GQ, DZ	MOL000098	quercetin	46.43	0.28	93
DZ3	DZ	MOL000422	kaempferol	41.88	0.24	34
C1	GQ, DZ, SZY	MOL000358	beta-sitosterol	36.91	0.75	18
GQ8	GQ	MOL008400	glycitein	50.48	0.24	17
DZ21	DZ	MOL002773	beta-carotene	37.18	0.58	17
SZY9	SZY	MOL008457	Tetrahydroalstonine	32.42	0.81	13
DZ23	DZ	MOL011604	Syringetin	36.82	0.37	12
DZ22	DZ	MOL008240	(E)-3-[4-[(1R,2R)-2-hydroxy-2-(4-hydroxy-3-methoxy-phenyl)-1-methylol-ethoxy]-3-methoxy-phenyl]acrolein	56.32	0.36	12
SY3	SY	MOL000322	Kadsurenone	54.72	0.38	11
DZ13	DZ	MOL009015	(-)-Tabernemontanine	58.67	0.61	11
SY8	SY	MOL000546	diosgenin	80.88	0.81	10
SY4	SY	MOL005430	hancinone C	59.05	0.39	9
DZ8	DZ	MOL007059	3-beta-Hydroxymethyllenetanshiquinone	32.16	0.41	8
B1	SDH, SY, SZY, GQ	MOL000449	Stigmasterol	43.83	0.76	8

DL = drug-likeness, OB = oral bioavailability, ONFH = osteonecrosis of the femoral head, YGY = You-Gui-Yin.

### 3.5. PPI network construction

**Figure 4.**
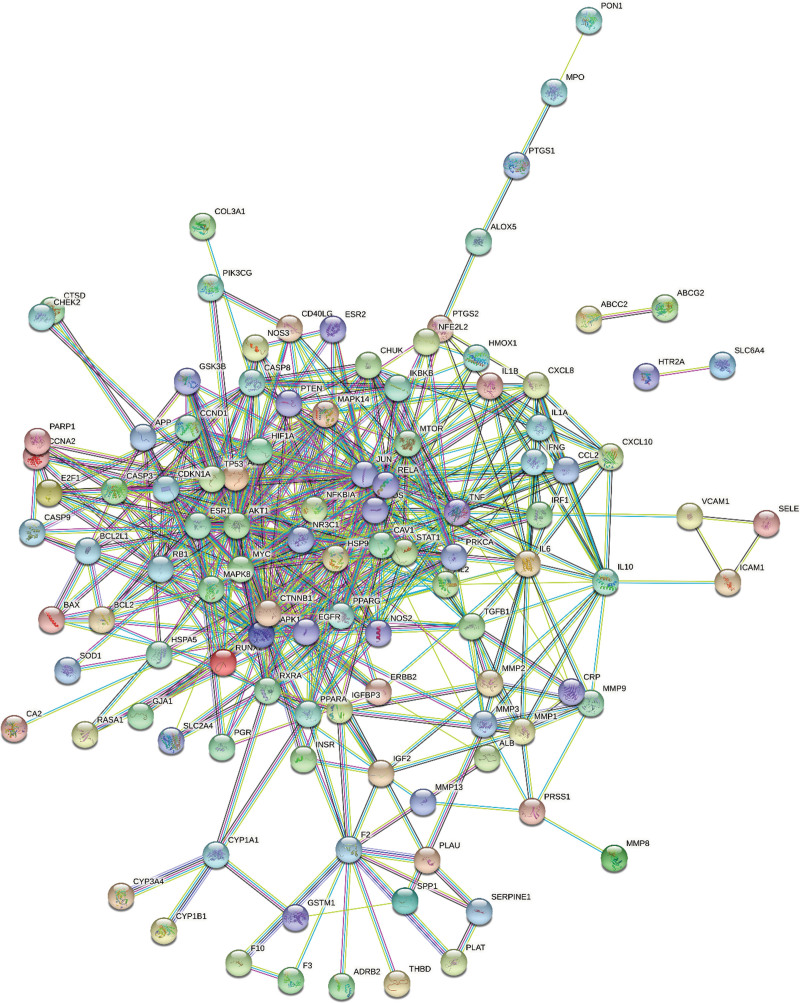
PPI Network of the 117 intersecting targets. PPI = protein-protein interaction.

After obtaining the 117 intersecting targets using the Venny online mapping platform between the targets of YGY and ONFH, The STRING 11.5 was used to construct the PPI network of the intersecting targets of YGY as related to the treatment of ONFH. After hiding the isolated nodes, the PPI network contained 111 nodes and 510 edges (Fig. 4). Target proteins with the top 14-degree values were selected as the core target proteins in the PPI network (Table 3).

**Table 3:** the core target proteins of YGY in the treatment of ONFH.

Target	Degree	Betweenness centrality (BC)	Closeness centrality (CC)
ALB	98	0.060887	0.865672
AKT1	97	0.042787	0.859259
TNF	95	0.032800	0.846715
IL6	94	0.032056	0.840580
TP53	91	0.023685	0.822695
IL1B	89	0.022606	0.811189
CASP3	86	0.017614	0.794521
JUN	83	0.014367	0.778523
MMP9	83	0.020380	0.778523
EGFR	80	0.022044	0.763158
PTGS2	80	0.011026	0.763158
HIF1A	79	0.010102	0.758170
CTNNB1	79	0.027305	0.758170
MYC	78	0.013485	0.753247

ONFH = osteonecrosis of the femoral head, YGY = You-Gui-Yin.

### 3.6. GO and KEGG pathway enrichment analysis results

**Figure 5.**
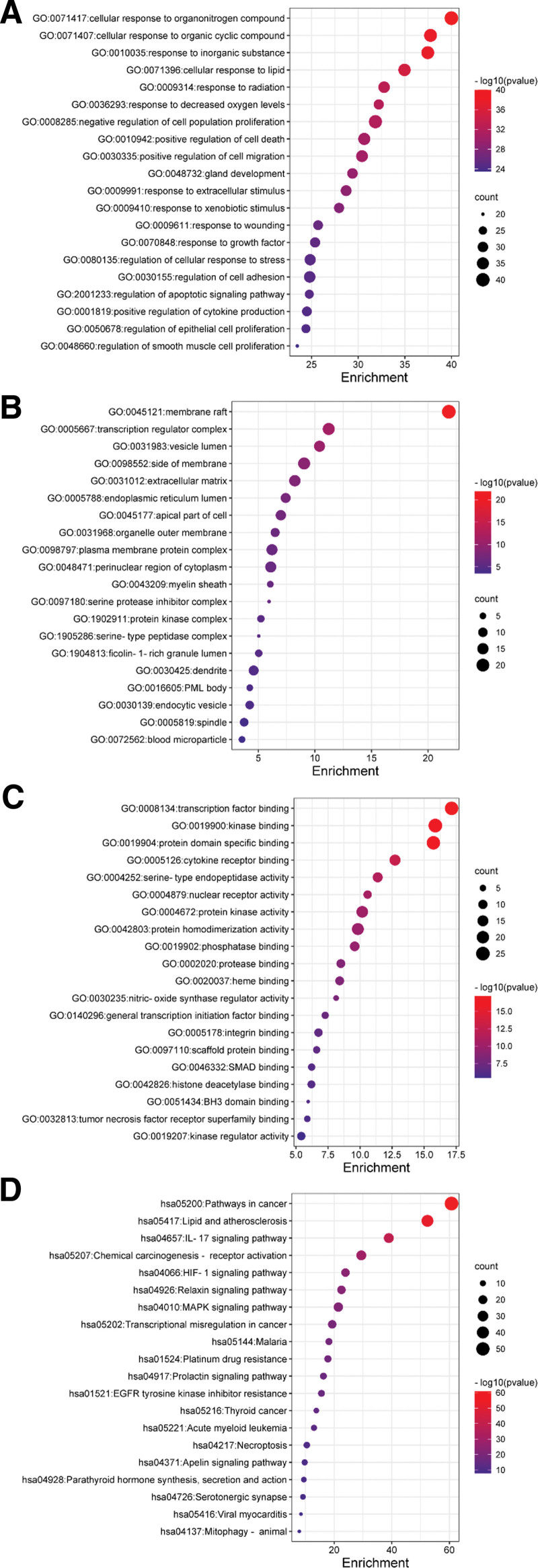
Results of GO functional enrichment analysis and KEGG signaling pathway enrichment analysis of the intersecting targets. (A) Biological process; (B) cellular component; (C) molecular function; (D) KEGG signaling pathway analysis. GO = Gene Ontology, KEGG = Kyoto Encyclopedia of Genes and Genomes.

**Figure 6.**
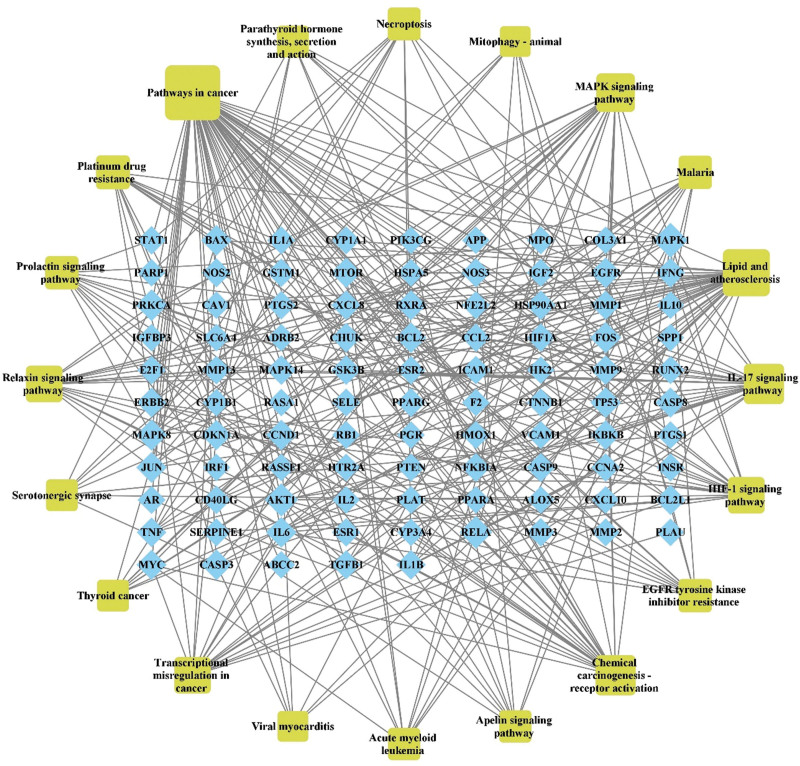
The target- KEGG signaling pathway network of YGY in the treatment of ONFH. The blue rhombic nodes represent the target genes, the yellow square nodes denote the signaling pathways. KEGG = Kyoto Encyclopedia of Genes and Genomes, YGY = You-Gui-Yin, ONFH = Osteonecrosis of the femoral head.

**Figure 7.**
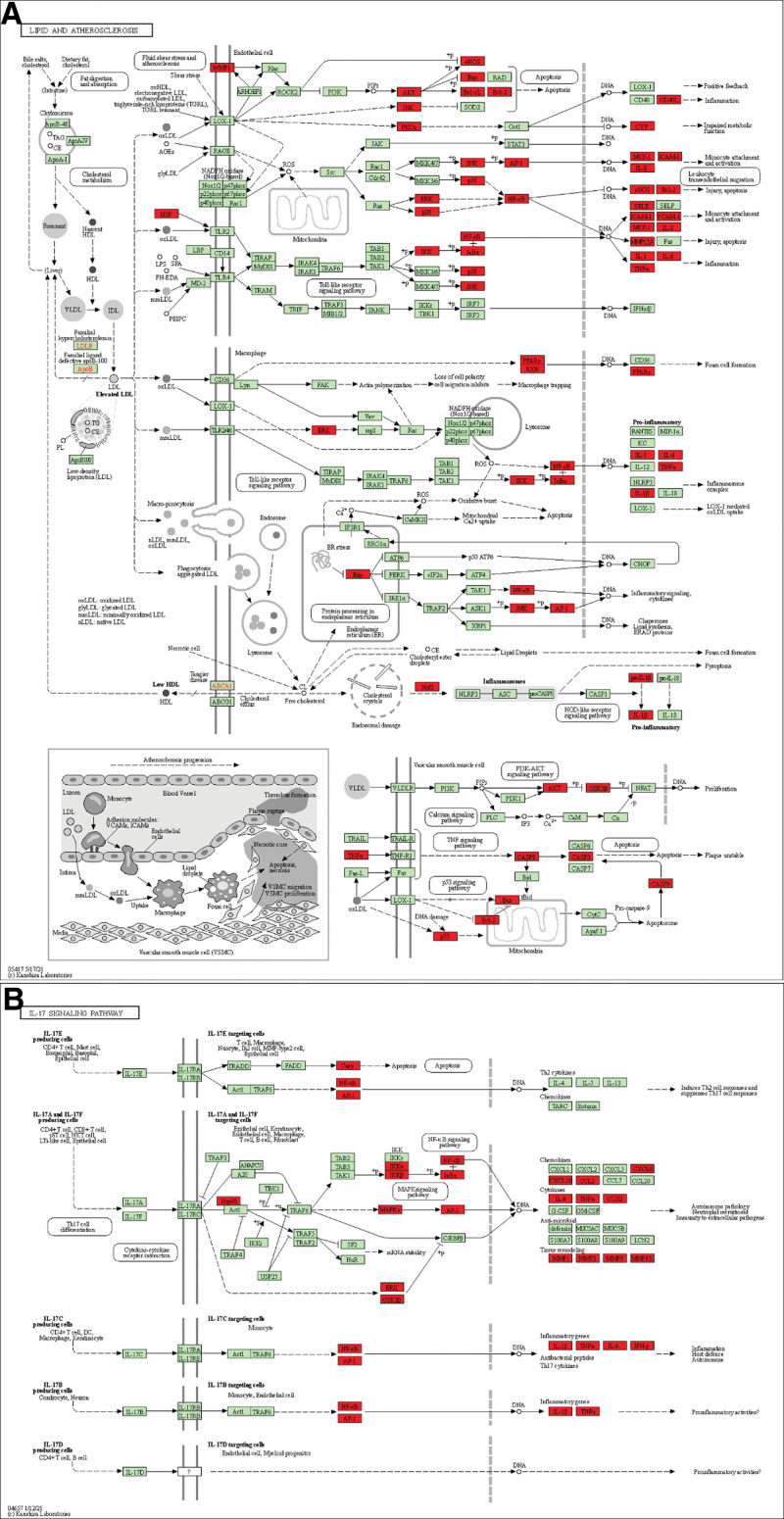
(A) Lipid and atherosclerosis signaling pathway adapted from KEGG (ID: hsa05417); (B) IL-17 signaling pathway adapted from KEGG (ID: hsa04657). The targets and genes of YGY for the treatment of ONFH were marked in red. KEGG = Kyoto Encyclopedia of Genes and Genomes, YGY = You-Gui-Yin, ONFH = Osteonecrosis of the femoral head.

To further clarify relevant functions and pathways, GO functional enrichment analysis and KEGG signaling pathway enrichment were performed for above 117 core targets using Metascape database platform. GO functional enrichment analysis was annotated in the following 3 categories, including BP, CC, and MF, with *P* < .01 serving as the threshold. To draw the GO functional heat maps of YGY in ONFH treatment, we selected the top 20 BP, CC, and MF results at *P* < .01 (Fig. 5A, 5B, and 5C). There were 1762 biological processes involved, including cellular response to organonitrogen compound, cellular response to organic cyclic compound, response to inorganic substance, cellular response to lipid, etc. CC analysis obtained a total of 94 enrichment items including membrane raft, transcription regulator complex, vesicle lumen, side of membrane, etc. One hundred and thirty-eight items were obtained via MF enrichment analysis, including transcription factor binding, kinase binding, protein domain specific binding, cytokine receptor binding, etc. KEGG signaling pathway analysis was done to elucidate the signaling pathways regulated by the therapeutic target genes. Results revealed that these target genes were distributed in 187 pathways, and the top 20 pathways were selected to construct a heat map (Fig. 5D). We also analyzed the association of the top 20 pathways with their corresponding target genes by constructing the target- KEGG signaling pathway network (Fig. 6). There were 115 nodes (including 20 pathways and 95 target genes) and 338 lines. The 95 blue rhombic nodes represented the target genes related to 20 signaling pathways, the 20 yellow square nodes denoted the signaling pathways. The targets and genes in the Lipid and atherosclerosis signaling pathway and IL-17 signaling pathway were shown in Figure 7A and 7B as examples.

### 3.7. Molecular docking results and analysis

**Figure 8.**
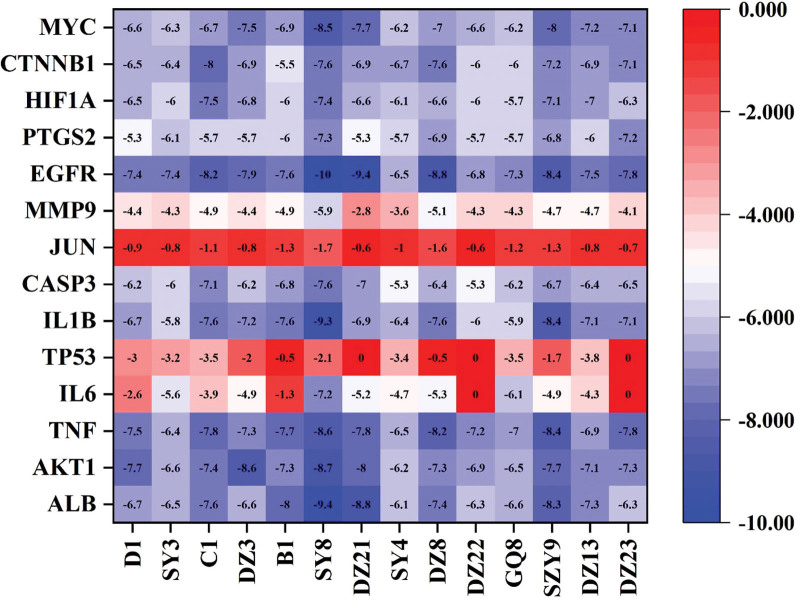
The heat map of molecular docking scores.

**Figure 9.**
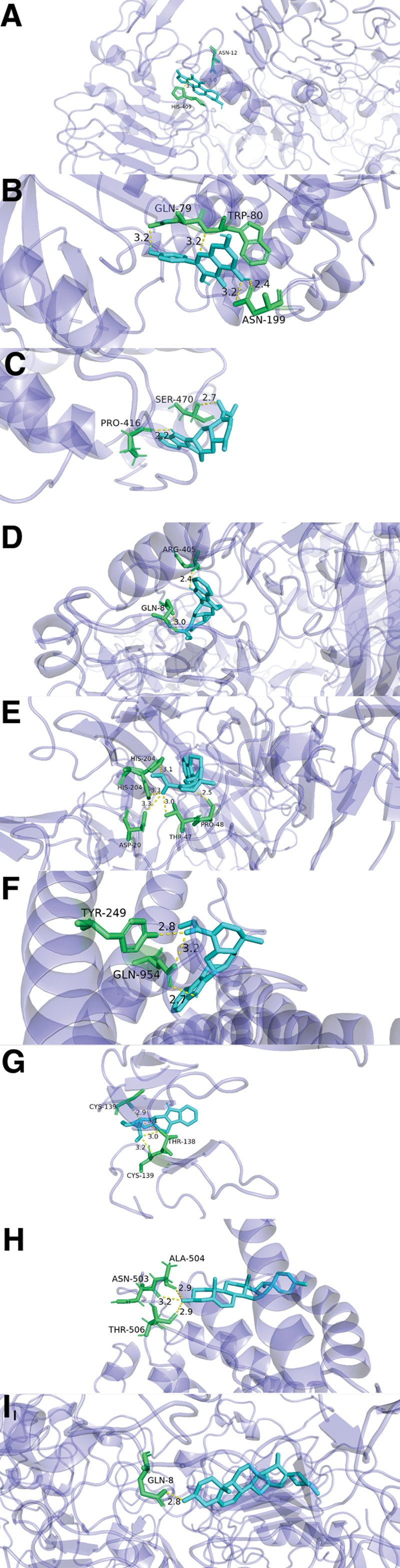
Molecular docking diagrams of key effective components to core target proteins. (A) EGFR-3-beta-Hydroxymethyllenetanshiquinone; (B) AKT1-kaempferol; (C) ALB-Tetrahydroalstonine; (D) EGFR-Tetrahydroalstonine; (E) IL1B-Tetrahydroalstonine; (F) MYC-Tetrahydroalstonine; (G) TNF--Tetrahydroalstonine; (H) ALB-diosgenin; (I) EGFR-diosgenin.

Molecular docking was performed among 14 key effective components selected from TCM-Component-Target-ONFH Network and 14 core target proteins selected from the PPI network using the Autodock Vina software (Fig. 8). When the affinity score value is less than −5 kcal/mol, the ligand is regarded to bind well to the receptor. The molecular docking results revealed that 75% of the affinity score values were lower than −5. The components with the lowest score values were kaempferol, diosgenin, Tetrahydroalstonine, beta-carotene, and 3-beta-Hydroxymethyllenetanshiquinone. The target proteins with the lowest score values were AKT1, ALB, EGFR, IL1B, MYC, and TNF. Part of the molecular docking models were illustrated in Figure 9. These findings indirectly verified that YGY had a regulatory effect on ONFH targets.

## 4. Discussion

Just as *Inner Canon of the Yellow Emperor* says, “The kidney stores essence and leads bone to generate marrow,” “Bone, the house of marrow,” “Marrow, the filling of bone,” “kidney produces the bone marrow.” According to *Yi Jing Jing Y*i, “Kidney stores essence, essence gives birth to marrow, marrow gives birth to bone, so kidney is the master of bone. The kidney produces marrow, and the marrow is sufficient if the essence is sufficient. The marrow exists in bone, and sufficient marrow makes the bone strong.”^[12]^ Thus, based on these theories in TCM, the kidney-tonifying herbs have the effects of generating marrow and strengthening bone. YGY in this study is from Jingyue Complete Works. It is a classic recipe which is a complexity formulation just like the Chinese patent drug called You-Gui-Wan (YGW) composed of ten TCM according to *Chinese Pharmacopeia 2020 version*. Most of the TCM components of YGW and YGY are the same which makes the 2 have a stable and close association, but the preparation methods are different. YGW is a Chinese patent medicine in pill form, while YGY is a Chinese medicine prescription in decoction form. The drug efficacies of Chinese medicine pills and Chinese medicine decoction are usually similar, but the drug efficiency of pills is milder and the action time is longer, while the drug efficiency of decoction is stronger, faster, and the action time is shorter. Despite the differences, both YGY and YGW can warm up and tonify kidney-Yang and correct the “kidney-Yang deficiency” state clinically.^[3,4,13,14]^ YGW and YGY were commonly used to treat gynecological diseases of kidney-Yang deficiency syndrome such as polycystic ovary syndrome,^[15]^ vaginal atrophy,^[16,17]^ infertility^[18]^ and premature ovarian failure.^[19]^ ONFH is also be differentiated by traditional Chinese medicine as kidney-Yang deficiency syndrome.^[5]^ Therefore, the underlying mechanism of YGY in treating ONFH is also to treat kidney-Yang deficiency syndrome. Some studies have shown that bone loss and osteoporosis occur in mice after ovary removal, and postmenopausal ovarian function decline is likely to lead to postmenopausal osteoporosis. All these indicated that the female reproductive system especially the ovary is closely related to bone, and YGY can inhibit bone loss by promoting osteogenic proliferation and differentiation and attenuating osteoclastogenesis.^[20,21]^ In western medicine, ONFH is a disease involving changes in local stem cell activity of the femoral head.^[22]^ The researchers found a decrease in the number, activity, or osteogenic differentiation of mesenchymal stem cells in the bone marrow in patients with ONFH.^[23,24]^ There was a close corresponding relationship between kidney essence and stem cells, and the decrease of the number and activity of stem cells happened in patients with kidney-Yang deficiency.^[25]^ The kidney-tonifying herbs could activate stem cells, increase the quantity of the stem cells,^[26]^ and adjust the microenvironment to a stable and coordinated state.^[27]^ Previous studies have shown YGY can effectively treat ONFH.^[6–8]^

Based on the network pharmacological research method, a total of 14 key effective components with the highest degree values including quercetin, kaempferol, beta-sitosterol, glycitein, beta-carotene, tetrahydroalstonine, syringetin, (E)-3-[4-[(1R,2R)-2-hydroxy-2-(4-hydroxy-3-methoxy-phenyl)-1-methylol-ethoxy]-3-methoxy-phenyl]acrolein, kadsurenone, (-)-tabernemontanine, diosgenin, hancinone C, 3-beta-Hydroxymethyllenetanshiquinone, stigmasterol in the treatment of ONFH were obtained in our study. Quercetin is a kind of natural polyphenolic flavonoid, and has been documented to possess the anti-inflammatory, cytoprotective, genoprotective and antioxidant effects in animal experimental models.^[28–31]^ In preclinical models of rheumatoid arthritis, gouty arthritis and osteoarthritis, quercetin showed significant joint protective effects,^[32]^ attenuated the synovial inflammation and hyperplasia, reduced the bone/cartilage destruction in joints, and decreased the secretion of inflammatory cytokine.^[33]^ Quercetin can also inhibit the macrophage polarization and the differentiation of osteoclasts, so that inhibit the inflammatory response, osteolysis, and osteonecrosis.^[34]^ Kaempferol is a flavonoid chemical with a wide range of medicinal properties such as anti-inflammatory, analgesic and anticancer effects.^[35]^ In vitro and in vivo experimental models, kaempferol have been acknowledged to have the bone-protective effect by inhibiting inflammation, oxidative stress, adipogenesis, osteoclastic differentiation, osteoclastic autophagy and osteoblastic apoptosis while activating osteoblastic autophagy, and to maintain the bone homeostasis and prevent resorption, loss and necrosis of bone.^[36,37]^ Beta-sitosterol is a kind of phytosterol, which has the effect of immune-regulation on macrophages and attenuates the inflammation reaction in mice model of rheumatoid arthritis.^[38]^By studying the effects of β-sitosterol on pleurisy, foot swelling and ear edema in mice, β-sitosterol was found to have anti-inflammatory effects.^[39]^And β-sitosterol can play an anti-inflammatory role by inhibiting the activation the inflammatory body NLRP3 in epidermal cells and macrophages to inhibit the production of CAS1 and the activation of MAPK signaling pathway, resulting in the significant decrease of of TNF-α, IL-1β, IL-6, and IL-8.^[40]^In addition, β-sitosterol also has antioxidant effect by reacting with organic acid to generate derivatives and inhibit the overexpression of TLR4 and NF-κB.^[41]^Therefore, β-sitosterol may be play a role in the treatment of femoral head necrosis through its anti-inflammatory, antioxidant effect. There were studies showed that glycitein suppressed osteoclast generation and induced osteoclast apoptosis in vitro and might also exert bone beneficial effects in vivo.^[42]^ Glycitein can also promote the differentiation of osteoblasts from their progenitor.^[43]^ β-carotene is a natural anti-oxidant, and can suppress osteoclast formation and bone resorption by inhibiting NF-κB signaling pathway.^[44]^Syringetin, a flavonoid derivative, can increase BMP-2 synthesis and subsequently activate SMAD1/5/8 and ERK1/2, and this effect may help to induce the maturation and differentiation of osteoblasts, thereby increasing bone mass.^[45]^Diosgenin, a steroidal sapogenin, can increase the formation of calcium deposits and enhance bone formation by stimulating the synthesis and secretion of Type 1 collagen, ALP, Runx2 and osteopontin expression.^[46]^ Folwarczna J. et al showed that Diosgenin increased compact bone formation and probably inhibited cancellous bone resorption, which led to improvement of mechanical properties of compact and cancellous bone.^[47]^ Stigmasterol has good pharmacological effects on antioxidant and anti-inflammatory.^[48,49]^ Chen WP et al found that Stigmasterol significantly inhibited the expression of matrix metalloproteinases and inhibited the degradation of cartilage.^[50]^ All the results of the above researches suggested that quercetin, kaempferol, beta-sitosterol, glycitein, beta-carotene, syringetin, diosgenin and stigmasterol had the potential effect in the treatment of ONFH. Other 6 components of the 14 major active components are also closely related to the key target genes associated with ONFH, and may also play important roles in the treatment of ONFH, which should be paid attention to and further studied.

Target proteins with the top 14-degree values were screened as the core target proteins in the treatment of ONFH in our study. AKT1, a protein kinase, is a major mediator of angiogenic signaling, which have shown the important role in bone vascularization and trabecular bone formation.^[51]^ It is also a unique signaling intermediate in osteoblasts that can regulate both the differentiation and formation of osteoblasts and osteoclasts to maintain bone mass.^[52]^ In addition, AKT1 contributes to the angiogenesis and ossification after injury at the end stage of endochondral bone formation, which might be helpful to the treatment of ONFH.^[53]^ EGFR, epidermal growth factor receptor, plays an important role in bone formation. There was a study showed that mice deficient in EGFR were growth retarded and exhibited severe bone defects, and signaling via EGFR stimulated osteoblast proliferation and inhibited their differentiation.^[54]^ Another research showed that down-regulated EGFR signaling leaded to the senescence of osteoprogenitors and the decline in bone formation on the endosteal surface of cortical bone.^[55]^ Stem cell function, a key contributor of bone remodeling, decreases in the process of ONFH and serum ALB is an effective activator of endogenous progenitor cells, so that ALB can successfully activate stem cell activity, support faster and functionally superior bone regeneration which is helpful for the treatment of ONFH.^[56]^ IL1B is a crucial inflammatory cytokine, can regulate the differentiation and activation of osteoclasts involved in the process of local bone erosiona, systemic osteoporosis and functional disabilities.^[57]^ Another study also showed that IL1B gene influenced the genetic susceptibility of steroid-induced ONFH.^[58]^ MYC, a highly efficient transcription factor, can promote the formation and differentiation of osteoclasts.^[59]^ Ni F. et al found that via the SLC7A5/c-MYC pathway IL-18 promoted the osteogenic differentiation of hBMSCs, which showed that MYC was also an important factor in osteogenic differentiation.^[60]^ TNF-α can regulate the early development of ONFH by mediating osteoblast autophagy and apoptosis^[61]^ and the onset of steroid-induced osteonecrosis of femoral head is influenced by TNF-α and hypoxia history,^[62]^ so TNF is an important target of ONFH. IL6 is an important cytokine and ONFH is accompanied by significant inflammatory response, so IL6 is an important target in the process of ONFH. There was a study showed that anti-IL6 therapy decreased hip synovitis and osteoclastic bone resorption and increased new bone formation after ischemic osteonecrosis.^[63]^ p53 protein, the product of TP53 gene, is a tumor suppressor that inhibits the growth of aberrant cells, sense and repair the damage of DNA.^[64]^ P53 can significantly co-regulate the mitophagy in BMSCs, decrease the accumulation of damaged mitochondria in cells, resist stress-induced apoptosis and senescence, and improve the repair of early steroid-induced ONFH.^[65]^

The KEGG enrichment analysis in our study indicated that the main signaling pathways involved in the treatment of ONFH by YGY are lipid and atherosclerosis signaling pathway, IL-17 signaling pathway, HIF-1 signaling pathway, relaxin signaling pathway, MAPK signaling pathway, and so on. Lipid and atherosclerosis signaling pathway participates in many physiological processes associated with lipid metabolism and atherosclerosis. Increased lipids followed by dyslipidaemia in human blood lead to increased blood viscosity and slow blood flow, which is prone to the formation of small emboli, atherosclerosis and blockage of blood vessels. Therefore, blood flow to the femoral head can be reduced or even interrupted, resulting in ONFH. Thrombophilia closely associated with lipid and atherosclerosis signaling pathway has been implicated as a potential cause of Legg-Perthes disease which is caused by ischemia of the ONFH in children.^[66]^ The lipoprotein level of >30 mg/dL was found in 16% of the Legg-Perthes disease group, which indicated the risk for atherosclerosis.^[66]^ Zhang Q. et al found that hyperlipidemia, fat hypertrophy, fat deposition within the femoral head intramedullary tissue, and fat embolism might cause ischemia by elevating intraosseous pressure and decreasing blood flow, eventually leading to ONFH. And dyslipidaemia also leads to a hypercoagulable state and aggravates ischemia in the process of ONFH.^[67]^ Zeng X. et al reported that avascular necrosis of the femoral head was significantly associated with blood lipid abnormalities in elderly patients with low-energy femoral neck fractures.^[68]^ Therefore, lipid and atherosclerosis signaling pathway is a very important pathway in the treatment of ONFH by YGY. IL-17, a kind of inflammatory cytokines, can stimulate macrophages and monocytes to produce proinflammatory factors and is mainly produced by T cells and monocytes. Through IL-17 signaling pathway, IL-17 performs the key function in regulating inflammatory processes in the inflamed synovium and peripheral blood in ONFH, which contributes to the development of ONFH.^[69]^ The relaxin signaling pathway is a potent stimulator of osteoclastogenesis from hematopoietic precursors, which regulate the activity of mature osteoclasts.^[70]^ Hypoxia and blood supply disruption are etiologies for ONFH, Hypoxia-inducible factor 1α (HIF-1α) is a master regulator of cellular response to hypoxia.^[71]^ And HIF-1α also plays a critical role in bone development, and contributes to regulate the bone regeneration and angiogenesis.^[72]^ HIF-1α, VEGF as well as apoptotic genes in the HIF-1α signaling pathway participate in the process of ischemic osteonecrosis.^[73]^The HIF-1α signaling pathway plays a vital role in regulating the processes of osteoclast activation, bone loss and angiogenesis,^[74]^ and is closely associated with the treatment of ONFH in many previous studies.^[75–77]^Via the HIF-1α/β-catenin pathway, hypoxia can stimulate angiogenesis and bone regeneration in BMSCs and contribute to treat the early stage of ONFH.^[78]^ In addition, IL-6 is produced in the hypoxic articular cartilage in the process of ONFH through HIF-1 signaling pathway, which can stimulate the inflammatory cytokine responses in synovial cells and develop the disease.^[79]^MAPK signaling pathway can activate NF-κB to regulate osteoblast autophagy and apoptosis in ONFH.^[61]^Activation of the MAPK signaling pathway can regulate osteoblast differentiation and inflammatory response,^[80]^ and can also reverse lipopolysaccharide – induced inhibition of osteoblast differentiation and bone resorption,^[81]^ so that promote the bone healing.

The potential binding activity and interaction of key effective components and core target proteins were further elaborated through molecular docking analysis in our study, which provided a reference for the subsequent research and development of targeted drugs. According to Figure 8, the molecular docking results revealed that 75% of the affinity score values were lower than -5, which indicated core effective components of YGY had strong binding activity with key target proteins. The images of molecular docking in Figure 9 showed the binding mode between the core effective components and key target proteins (AKT1, ALB, EGFR, IL1B, MYC, and TNF) as well as the interaction with the surrounding amino acid residues. The hydrophobic force and hydrogen bond in molecular docking results confirmed the potential therapeutic mechanism of YGY, showing excellent binding activity with target proteins in ONFH. These all suggest that YGY may play its pharmacological role by regulating the key targets in ONFH.

However, there are still limitations in this study. Firstly, there is less literature and lack of information on the TCM study in public databases such as PubMed. Therefore, there is selection bias in this study which leads to underestimate the usage of YGY. Secondly, network pharmacology and molecular docking are only the preliminary exploration of the possible mechanisms, the prediction results of our study should be further verified by animal experiments and clinical trials.

## 5. Conclusion

According to the results in our study, quercetin, kaempferol, beta-sitosterol, glycitein, beta-carotene, tetrahydroalstonine, syringetin, (E)-3-[4-[(1R,2R)-2-hydroxy-2-(4-hydroxy-3-methoxy-phenyl)-1-methylol-ethoxy]-3-methoxy-phenyl]acrolein, kadsurenone, (-)-tabernemontanine, diosgenin, hancinone C, 3-beta-Hydroxymethyllenetanshiquinone, stigmasterol are the key effective components, and ALB, AKT1, TNF, IL6, TP53, IL1B, CASP3, JUN, MMP9, EGFR, PTGS2, HIF1A, CTNNB1, and MYC are the core target proteins in the treatment of ONFH by YGY. The main signaling pathways involved in the treatment of ONFH by YGY are Lipid and atherosclerosis signaling pathway, IL-17 signaling pathway, HIF-1 signaling pathway, relaxin signaling pathway, MAPK signaling pathway, and so on. The study is the first to explore the mechanism of YGY in the treatment of ONFH via strategies of network pharmacology and molecular docking. And it reveals that YGY can treat ONFH through multicomponents, multitargets, and multipathways, which provides a reference for the subsequent research, development of targeted drugs and clinical application.

## Author contributions

**Conceptualization:** Zhi-Yuan Yao.

**Data curation:** Shu-Yao Fan, Zhou-Feng Song, Zhan-Chun Li.

**Investigation:** Zhan-Chun Li.

**Methodology:** Shu-Yao Fan, Zhou-Feng Song, Zhan-Chun Li.

**Project administration:** Zhi-Yuan Yao.

**Validation:** Shu-Yao Fan.

**Visualization:** Shu-Yao Fan, Zhou-Feng Song, Zhan-Chun Li.

**Writing – original draft:** Zhi-Yuan Yao.

**Writing – review & editing:** Zhi-Yuan Yao.
